# IDLDA: An Improved Diffusion Model for Predicting LncRNA–Disease Associations

**DOI:** 10.3389/fgene.2019.01259

**Published:** 2019-12-06

**Authors:** Qi Wang, Guiying Yan

**Affiliations:** ^1 ^Academy of Mathematics and Systems Science, Chinese Academy of Sciences, Beijing, China; ^2^School of Mathematical Sciences, University of Chinese Academy of Sciences, Beijing, China

**Keywords:** long non-coding RNA, disease, association prediction, computational prediction model, diffusion model

## Abstract

It has been demonstrated that long non-coding RNAs (lncRNAs) play important roles in a variety of biological processes associated with human diseases. However, the identification of lncRNA–disease associations by experimental methods is time-consuming and labor-intensive. Computational methods provide an effective strategy to predict more potential lncRNA–disease associations to some degree. Based on the hypothesis that phenotypically similar diseases are often associated with functionally similar lncRNAs and *vice versa*, we developed an improved diffusion model to predict potential lncRNA–disease associations (IDLDA). As a result, our model performed well in the global and local cross-validations, which indicated that IDLDA had a great performance in predicting novel associations. Case studies of colon cancer, breast cancer, and gastric cancer were also implemented, all lncRNAs which ranked top 10 in both databases were verified by databases and related literature. The results showed that IDLDA might play a key role in biomedical research.

## Introduction

Non-coding RNA (ncRNA) is a kind of RNA molecule that is not translated into protein (Bertone et al., 2004; Wilusz et al., 2009). In decades past, lncRNA was considered as transcriptional noise and few people studied it. Nowadays, accumulating evidence has proved the key regulatory role of lncRNAs in many significant biological processes (Esteller, 2011). For example, some mutated and dysfunctional lncRNAs were implicated in a lot of human diseases such as renal cancer (Meng et al., 2014; Xu et al., 2015), breast cancer (Barsyte-Lovejoy et al., 2006; Gupta et al., 2010), hepatocellular cancer (Calin et al., 2007; Panzitt et al., 2007), prostate cancer (De Kok et al., 2002; Széll et al., 2008), lung cancer (Ji et al., 2003; Zhang et al., 2003), colon cancer (Pibouin et al., 2002), leukemia (Calin et al., 2007)and cardiovascular diseases (Congrains et al., 2012). There are many well-known lncRNA-related biological databases such as NRED (Dinger et al., 2009), NONCODE (Liu et al., 2005; Xie et al., 2014; Zhao et al., 2016), LncRNADisease (Chen et al., 2013), Lnc2Cancer (Ning et al., 2016) and lncRNAdb (Quek et al., 2015), including the information about lncRNA and little lncRNA–disease associations.

Recently, exploiting potential lncRNA–disease associations have become a growing significant research area. Many associations between lncRNA and human diseases have been identified by medical experiments, but which is costly and time-consuming. Predicting potential associations by the mathematical method and computational inference for experimental verification is a quite certain well-selected alternative (Chen et al., 2017; Chen et al., 2019).

Chen and Yan (2013) presented the Laplacian Regularized Least Squares for LncRNA–Disease Association (LRLSLDA), which is a semi-supervised learning framework to identify potential associations by integrating known associations and lncRNA expression profiles. Liu et al. (2014) put forward a computational model to predict potential lncRNA–disease associations by integrating many types of data such as gene expression profiles, human lncRNA expression profiles, and human disease-associated gene data. Li J, et al. (2014) presented a prediction method based on genome location information to discover potential vascular disease-related lncRNAs. Sun et al. (2014) established a lncRNA functional similarity network and used the random walk model to predict potential lncRNA–disease associations. However, this method cannot be applied to the lncRNAs without any known associated diseases. Yang et al. (2014) also proposed a network-based method to identify lncRNA–disease associations. And Yang’s method had a great performance to predict lncRNA–disease associations but it did not take into account various similarities. Chen (2015a) constructed a Katz measure model (KATZLDA) to predict lncRNAs associated with diseases, especially isolated disease-related lncRNAs. However, the method relies excessively on a network topology structure. Ping et al. (2019) constructed a lncRNA–disease bipartite network to infer potential lncRNA–disease associations by integrating two similarity calculation methods for lncRNAs and diseases. Gao et al. (2019) developed a dual sparse collaborative matrix factorization method based on gaussian kernel function (DSCMF) to predict novel lncRNA–disease associations. They considered the sparsity of lncRNA–disease association and used the L2,1-norm to ensure its sparsity in optimization.

In this paper, we developed an improved diffusion model for predicting lncRNA–disease associations (IDLDA) based on the hypothesis that phenotypically similar diseases are often associated with functionally similar lncRNAs and *vice versa*. IDLDA achieved reliable predictions with global and local cross-validations and it obtained higher AUROC than some previously proposed methods. Our results showed that the predicted top 10 lncRNAs in both databases were confirmed by databases and literature, and there were only 2, 2, and 1 lncRNAs which ranked top 50 by IDLDA in both databases that were not confirmed. All these results demonstrated the effectiveness and value of IDLDA in identifying potential lncRNA–disease associations. Data and code are freely available for research purposes only, you can email the author for it.

## Materials and Methods

### Data Collection and Pre-Processing

LncRNADisease (Chen et al., 2013) and Lnc2Cancer (Ning et al., 2016) are two well-known databases that we can apply to extract known lncRNA–disease associations. We got 687 experimentally verified lncRNA-disease associations (**Supplementary Tables 1** and **3**) including 372 lncRNAs and 246 diseases in the LncRNADisease, and 1,102 experimentally verified lncRNA-disease associations (**Supplementary Tables 2** and **4**) including 667 lncRNAs and 97 cancers in the Lnc2Cancer. These datasets were utilized as not only the gold standard datasets in the cross-validation but also the training datasets in novel lncRNA–disease association prediction. In addition, we also combined the data from the two datasets to make a complete training data set for validation which named combined dataset. There are 1669 experimentally verified lncRNA–disease associations including 944 lncRNAs and 295 diseases. This dataset (**Supplementary material Data Sheet 1**) can better illustrate the credibility of the model. To the author’s knowledge, this is the first article to combine the data of these two databases for model validation.

We constructed lncRNA–disease associations as a bipartite graph *G*(*V*,*E*) as follows. *V*=*L*∪^​^ *D* is the vertex set, where *L* is the lncRNA set { *l*_1_,*l*_2_,…,*l* *_Nl_* }, *D* is the disease set { *d*_1_,*d*_2_,…,*d* *_Nd_* }, and denote the edge set *E*={ *e* *_ij_*:*d* *_i_*∈*D*,*l* *_j_*∈*L* }. *N* *_d_* and *N* *_l_* represent the number of diseases and the number of lncRNAs, respectively. Here, the lncRNA–disease association can be represented by an adjacency matrix *A*={*a* *_ij_*}*_Nd_*_×_ *_Nl_*, where *a* *_ij_*=1 if disease *d* *_i_* and lncRNA *l* *_j_* have experimentally validated relation in the databases, while the unknown associations are set to 0 indicating that they will be ranked.

**Figure 1:**
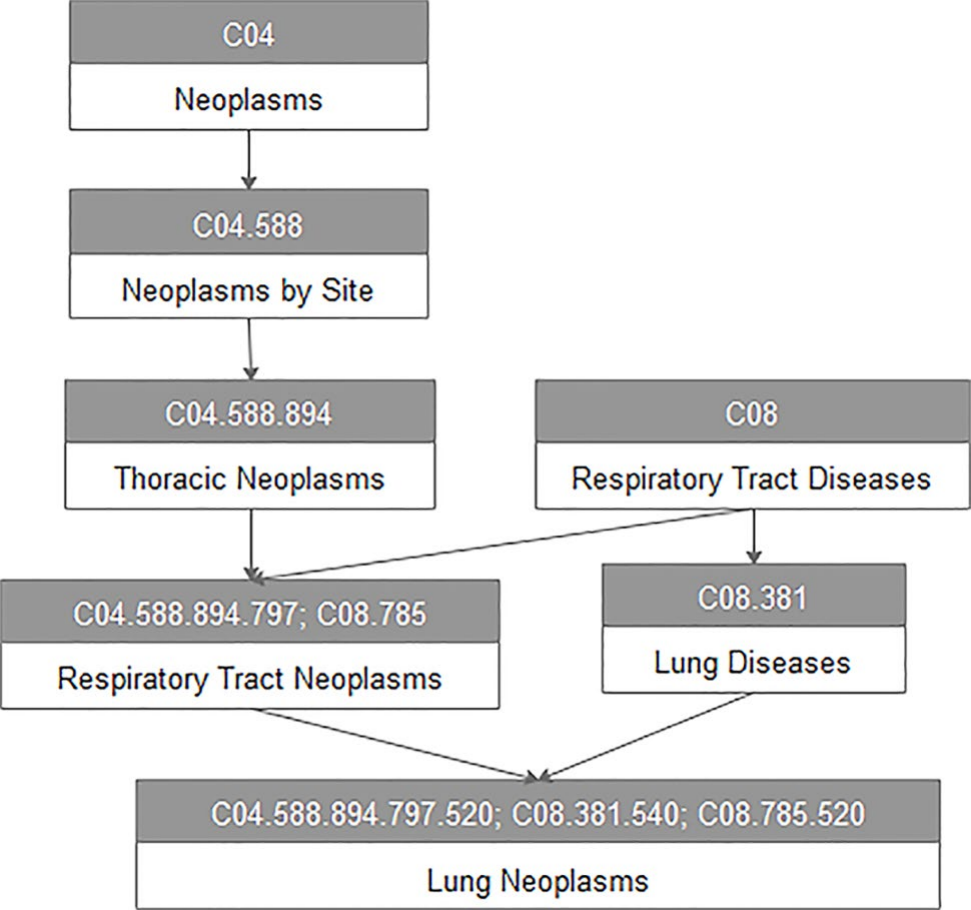
The disease DAG of lung neoplasms.

For every disease term *d* *_j_* in the MeSH database, we constructed a directed acyclic graph *DAG*(*d* *_j_*) based on the MeSH descriptors of Category C downloaded from the National Library of Medicine. For example, **Figure 1** represents the DAG of lung neoplasms. All vertices in the DAG are connected by a direct edge from a more general term, we call it parent, to a more specific term, and we call it child (Chen et al., 2015). Here, *V*(*DAG*(*d* *_j_*)) indicated the vertex set including vertex *d* *_j_* and its ancestor vertices, and *E*(*DAG*(*d* *_j_*)) was the edge set of corresponding direct links from a parent vertex to a child vertex, which represented the relationship between different diseases.

### Ensemble Similarity

#### Disease Ensemble Similarity

For a given disease *d* *_j_*, in the *DAG*(*d* *_j_*), the contribution of each disease semantic term *C* *_dj_*(*d* *_i_*) of disease *d* *_i_* was defined as follows (Wang et al., 2010):

(1) $$C_{d_{j}}(d_{i})=\{\begin{array}{ll} 1, & if\;d_{i}=d_{j}\\ \max\{∆\times C_{dj}(d_{k})|d_{k}\in children\;of\;d_{i}\}, & if\;d_{i}\neq d_{j} \end{array}$$

where Δ was a decay factor of semantic contribution, which should be between 0 and 1. According to some previous studies (Wang et al., 2010; Chen et al., 2015; Chen, 2015a), this value was 0.5 here. Accordingly, the contribution to the semantic value of disease *d* *_j_* itself was defined as 1. Meanwhile, the contribution of its ancestor disease should be multiplied by Δ.

According to this way to measure disease semantic similarity, we thought that two diseases *d* *_i_* and *d* *_j_* which had a larger *DAG*(*d* *_i_*) ∩^​^ *DAG*(*d* *_j_*) should have a higher semantic similarity. Thus, the semantic score of disease *d* *_j_* was acquired by adding up all the contributions from ancestor diseases and disease *d* *_j_* itself. Define the semantic score (*C*) of disease *d* *_j_* as follows:

(2) $$C(d_{j})=\Sigma_{d_{i}\in DAG(d_{j})}C_{d_{j}}(d_{i})$$

Thus, disease semantic similarity (*SS*) between disease *d* *_i_* and disease *d* *_j_* can be written as (Chen et al., 2018):

(3) $$SS_{ij}=\frac{\Sigma_{t\in V(d_{i})\cap^{}V(d_{j})}C_{d_{i}}(t)+C_{d_{j}}(t)}{C(d_{i})+C(d_{j})}$$

Based on the basic assumption that two lncRNAs with more functional similarity prefer to be more related to similar diseases and *vice versa* (Lu et al., 2008), we could obtain disease similarity by the topologic information of the known lncRNA–disease association network. Accordingly, we introduced the Gaussian interaction profile kernel for calculating the similarity between diseases as a part of the disease similarity (van Laarhoven et al., 2011; Chen and Yan, 2013). Then we utilized the following equation to obtain disease Gaussian kernel similarity (*KD*) between disease *d* *_i_* and disease *d* *_j_*.

(4) $$KD_{ij}=\exp(-\gamma_{d}||IP(d_{i})-IP(d_{j})|{|}^{2})$$

where *IP*(*d* *_i_*) was the *i*-th column of matrix *A*. The parameter *γ* *_d_* was a parameter for adjusting the bandwidth of the kernel, which should be updated by using a new bandwidth parameter $\gamma_{d}^{’}$ divided by the average value of the associations with lncRNAs for all diseases. According to the previous study (Cheng et al., 2012; Sun et al., 2016), $\gamma_{d}^{’}$ was set to 1 to control thekernel bandwidth.

Thus, *γ* *_d_* could be defined as follows:

(5) $$\gamma_{d}={\gamma^{\prime }}_{d}/\left(\frac{1}{N_{d}}\sum_{i=1}^{N_{d}}{\left||IP(d_{i})|\right|}^{2}\right)$$

Define the disease ensemble similarity (*DS*) between disease *d* *_i_* and disease *d* *_j_* as follows:

(6) $$DS_{ij}=\{\begin{array}{l} 1/2(SS_{ij}+KD_{ij}),SS_{ij}\neq 0\\ KD_{ij},\;SS_{ij}=0 \end{array}$$

#### LncRNA Ensemble Similarity

For a disease *d* *_i_* and a group of diseases *D*, their similarity score *S* between them was defined as (Chen et al., 2015):

(7) $$S(d_{i},D)=\underset{d_{j}\in D}{\max}SS_{ij}$$

Let *D*(*l* *_i_*) and *D*(*l* *_j_*) be the set of diseases related to lncRNA *l* *_i_* and lncRNA *l* *_j_*, respectively. Define similarity score *S* between *D*(*l* *_i_*) and *D*(*l* *_j_*) as follows:

(8) $$S\left(D\left(l_{i}\right),D\left(l_{j}\right)\right)=\sum_{t\in D\left(l_{i}\right)}S\left(t,D\left(l_{j}\right)\right)+\sum_{t\in D\left(l_{j}\right)}S\left(t,D\left(l_{i}\right)\right)$$

Usually, most of researchers believe that lncRNAs with similar functions are more likely related to similar diseases and *vice versa* (Yang et al., 2009; Chen and Yan, 2013; Liu et al., 2014; Sun et al., 2014; Yang et al., 2014; Chen et al., 2015; Chen, 2015a; Gu et al., 2017). Therefore, the functional similarity between lncRNA *l* *_i_* and lncRNA *l* *_j_* was calculated as follows:

(9) $$FS_{ij}=\frac{{\sum^{}}_{t\in D\left(l_{i}\right)}S\left(t,D\left(l_{j}\right)\right)+{\sum^{}}_{t\in D\left(l_{j}\right)}S\left(t,D\left(l_{i}\right)\right)}{\left|D\left(l_{i}\right)\right|+\left|D\left(l_{j}\right)\right|}$$

where | *D*(*l* *_i_*) | and | *D*(*l* *_j_*) | were the numbers of diseases associated with lncRNA *l* *_i_* and lncRNA *l* *_j_*, respectively.

Similarly, the Gaussian kernel similarity between lncRNA *l* *_i_* and lncRNA *l* *_j_* was defined as follows (van Laarhoven et al., 2011; Chen and Yan, 2013):

(10) $$KL_{ij}=\text{exp(}-\gamma_{l}||IP(l_{i})-IP(l_{j})|{|}^{2})$$

(11) $$\gamma_{l}={\gamma^{\prime }}_{l}/\left(\frac{1}{N_{l}}\sum_{i=1}^{N_{l}}||IP\left(l_{i}\right)|{|}^{2}\right)$$

where $\gamma_{l}^{’}$ = 1 (Cheng et al., 2012; Sun et al., 2016).

Define the lncRNA ensemble similarity (*LS*) between lncRNA *l* *_i_* and lncRNA *l* *_j_* as follows:

(12) $$LS_{ij}=\{\begin{array}{l} 1/2\left(FS_{ij}+KL_{ij}\right),FS_{ij}\neq 0\\ KL_{ij},\;FS_{ij}=0 \end{array}$$

### Ensemble Associations

On the basis of the ensemble similarity matrix *DS* and *LS*, we could obtain two ensemble associations *DA*={ *DA* *_ij_* }*_Nd_*_×_ *_Nl_* and *LA*={ *LA* *_ij_* }*_Nd_*_×_ *_Nl_*. *DA* *_ij_* and *LA* *_ij_* can be written as:

(13) $$DA_{ij}=\sum_{l=1}^{N_{d}}DS_{il}A_{lj}$$

(14) $$LA_{ij}=\sum_{l=1}^{N_{l}}A_{il}LS_{lj}$$

### An Improved Diffusion Model on the Network

We applied an improved diffusion model to calculate the information transmitted in the bipartite graph, which was quantified to solve the correlation between lncRNAs and diseases.

First of all, we selected one disease *D* *_u_* as seed, so the initial resources were located on each lncRNA, which associated with disease *D* *_u_*. Based on the hypothesis that lncRNAs with similar functions are usually related to similar diseases and *vice versa*. All the initial resources in *L* flowed to *D* by *LA* and *DA*. Thus, the comprehensive index (resources) of the *d* *_j_* vertex was shown as follows:

(15) $$g\left(d_{j}|d_{u}\right)=\alpha \sum_{i=1}^{N_{l}}\frac{LA_{ji}}{{\sum^{}}_{j=1}^{N_{d}}LA_{ji}}LA_{ui}+\left(1-\alpha \right)\sum_{i=1}^{N_{l}}\frac{DA_{ji}}{{\sum^{}}_{j=1}^{N_{d}}DA_{ji}}DA_{ui}$$

Each disease scattered the received resources to its associated lncRNAs, the resources located on the *d* *_j_* vertex returned back to *L* by *LA* and *DA*. Then the final comprehensive index (resources) of the *l* *_i_* vertex as shown below:

(16) $$IDLDA\_score\left(l_{i}|d_{u}\right)=\beta \sum_{j=1}^{N_{d}}\frac{LA_{ji}}{{\sum^{}}_{i=1}^{N_{l}}LA_{ji}}g\left(d_{j}|d_{u}\right)+\left(1-\beta \right)\sum_{j=1}^{N_{d}}\frac{DA_{ji}}{{\sum^{}}_{i=1}^{N_{l}}DA_{ji}}g\left(d_{j}|d_{u}\right)$$

**Figure 2:**
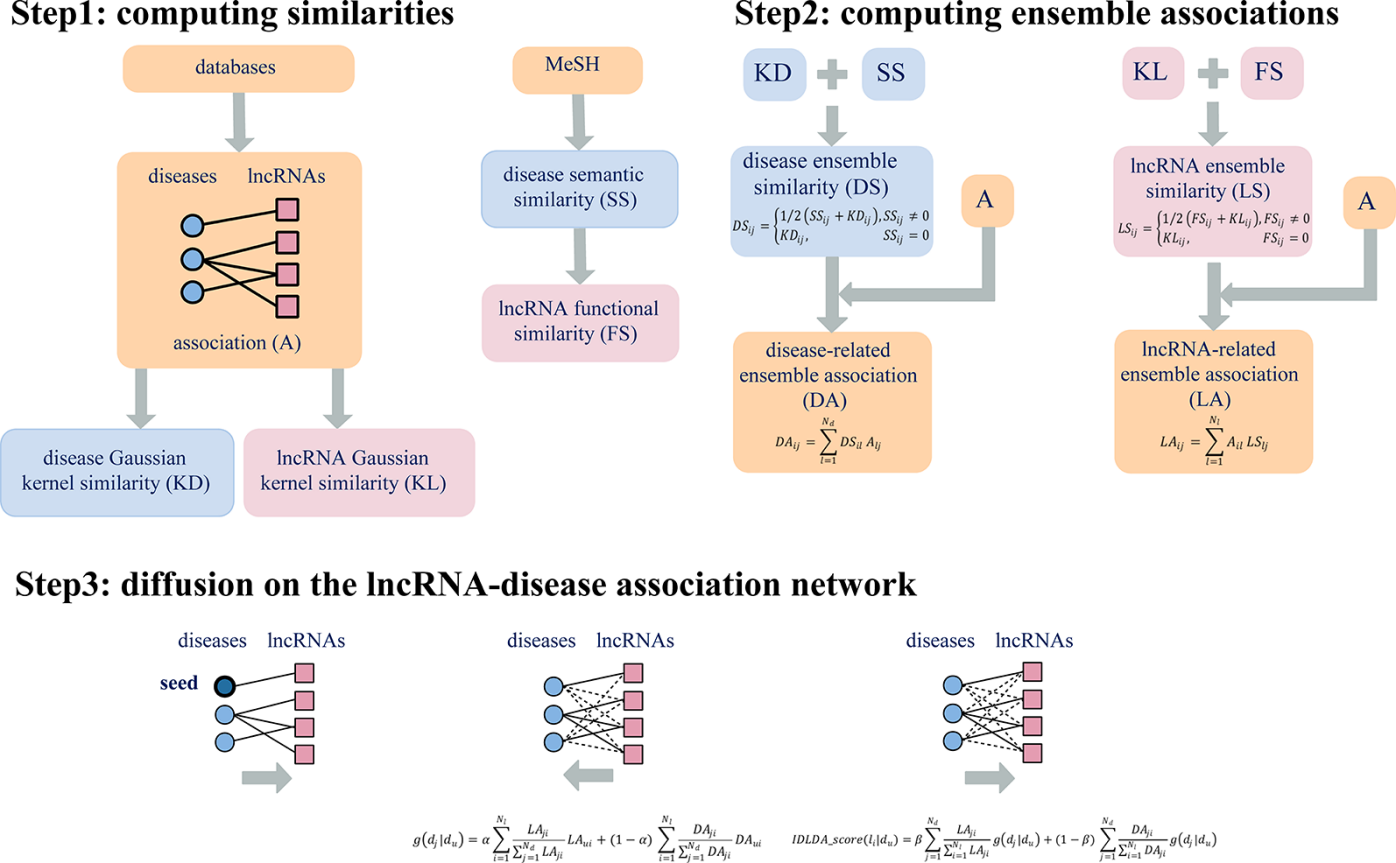
Flowchart of IDLDA. N_d_ and N_l_ represent the number of diseases and the number of lncRNAs, respectively.

Here the parameters α, β were used to balance the contribution between *LA* and *DA*. Therefore, for a given disease *D* *_u_*, we could obtain the comprehensive index IDLDA-score of every lncRNA. Accordingly, we got the predicted ranks of all lncRNAs for every disease. This predicted result can be represented by a rank matrix *R*={*r* *_ij_*}*_Nd_*_×_ *_Nl_*, where *r* *_ij_* indicated the relevance score between disease *d* *_i_* and lncRNA *l* *_j_*. The larger the value of *r* *_ij_*, the more likely disease *d* *_i_* and lncRNA *l* *_j_* are to be related. Thus, IDLDA can predict not only new disease-related lncRNAs but new lncRNA-related diseases. The flow chart of IDLDA is shown in **Figure 2**.

## Results

In this section, we first analyzed some properties of the lncRNA–disease association network. Next, we used global and local cross-validations and performed enrichment analysis to evaluate the performance of IDLDA. Then, we conducted case studies to verify the efficiency of IDLDA in discovering some potential disease-related lncRNAs.

### Properties of the lncRNA–Disease Association Network

We analyzed the lncRNA–disease association network’s characteristics to obtain a whole view of it (**Table 1**). Among them, density denotes the number of edges divided by the number of possible edges. As we can see from **Table 1**, there are very few associations available, so it is very important to predict potential associations.

**Table 1:** Global characteristics of the lncRNA–disease association.

	**No. of lncRNAs**	**No. of Diseases**	**No. of Associations**	**Density**
LncRNADisease	372	246	687	0.0075
Lnc2Cancer	667	97	1,102	0.0170
Combined dataset	944	295	1,669	0.0060

### Cross-Validation Tests

A receiver operating characteristic (ROC) curve is a graphical plot that shows the diagnostic ability of the binary classifier system because its recognition thresholds are different (Fawcett, 2006). AUROC (Area Under Receiver Operating Characteristic Curve) is the area under the ROC curve with a value between 0 and 1. AUROC can intuitively evaluate the quality of classifier, the larger the value, the better. The similarities between diseases and lncRNAs rely on known associations. Therefore, the disease ensemble similarity and lncRNA ensemble similarity should be recalculated in each repetition of the experiment. The IDLDA method had two parameters, i.e. α and β. Here, when the values of α and β took 0, 0.1, 0.2, …,1 the values in the leave-one-out cross-validation (LOOCV), the AUROC were calculated. The highest AUROC value was 0.9513 (α=0.3, β=0.5) in the combined dataset. As a result, the parameters (α, β) in the combined dataset was (0.3, 0.5).

Our model could predict not only new lncRNAs but also new diseases. Here, we adopt three cross-validations to evaluate the prediction accuracy of the model from global and local perspectives. The first cross-validation is LOOCV, some elements in the matrix A were randomly selected as the training set and the remaining elements as the test set; the second cross-validation is CVr, selected some rows of the matrix A randomly as the training set and the remaining data as the test set; the third cross-validation is CVc, selected some columns of the matrix A randomly as a training set and the remaining data as a test set.

**Figure 3:**
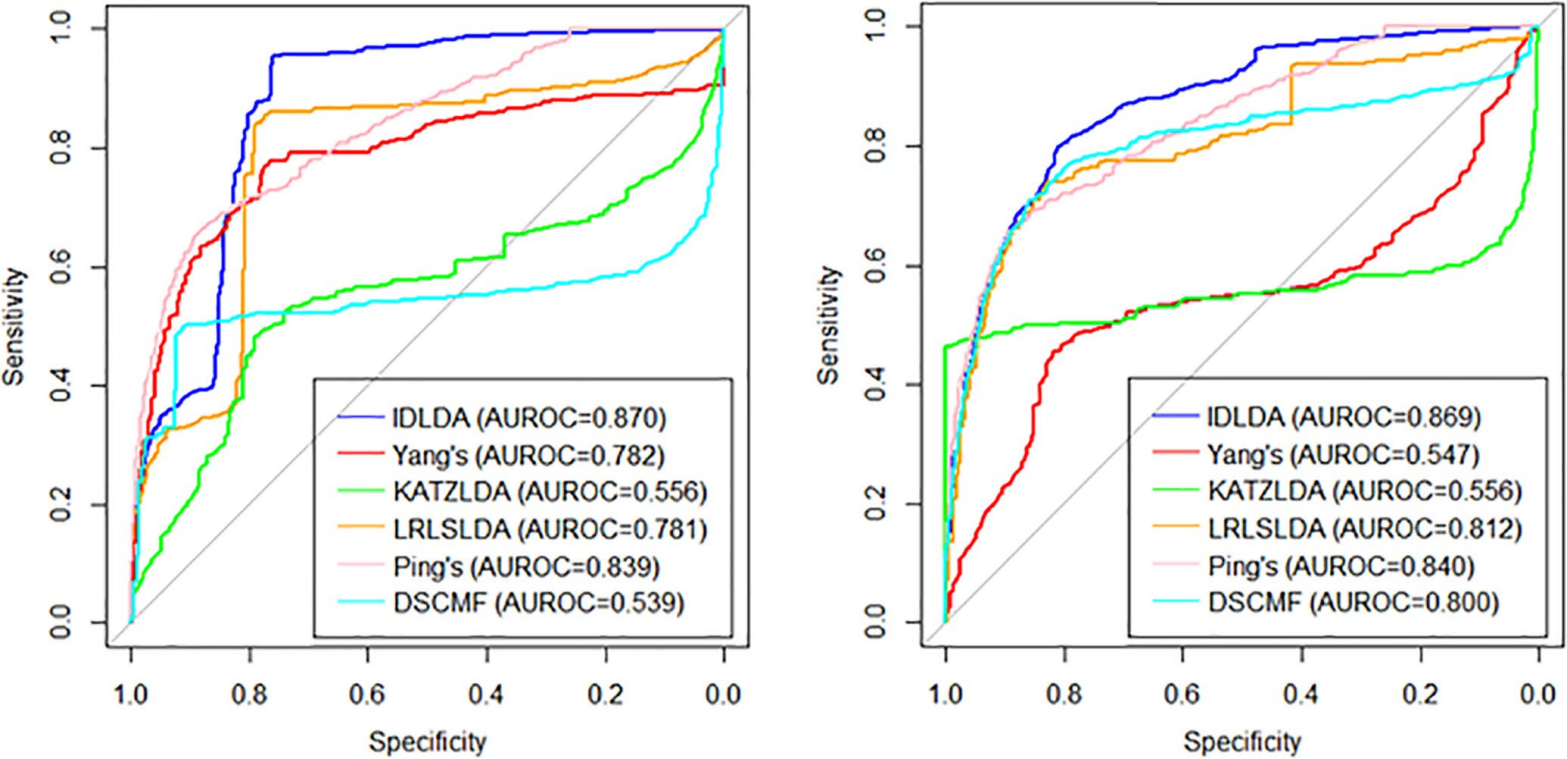
The ROC curves of the different methods with local cross-validation by row (Left) and by column (Right).

Among the three cross-validations, LOOCV was global cross-validation, which could test the prediction accuracy of the model on the original data set. For LOOCV, each known lncRNA–disease association was taken in turn as a testing sample and the remaining associations were used as training samples. And the baseline indicated random performance. In order to ensure the consistency of input data, the similarities of diseases and lncRNAs in other methods is consistent with the similarity of the IDLDA, which can better compare the predictive ability of the model itself. The AUROC of the combined dataset was 0.9513. We demonstrated that our approach significantly outperforms great performance (**Supplementary Table 5**). CVr and CVc were local cross-validations, which could test the prediction accuracy of the model for newly added diseases and lncRNAs respectively. The results of CVr (**Figure 3**, Left) and CVc (**Figure 3**, Right) showed that IDLDA had great performance in predicting novel lncRNA-related diseases and disease-related lncRNAs.

### Enrichment Analysis

**Figure 4:**
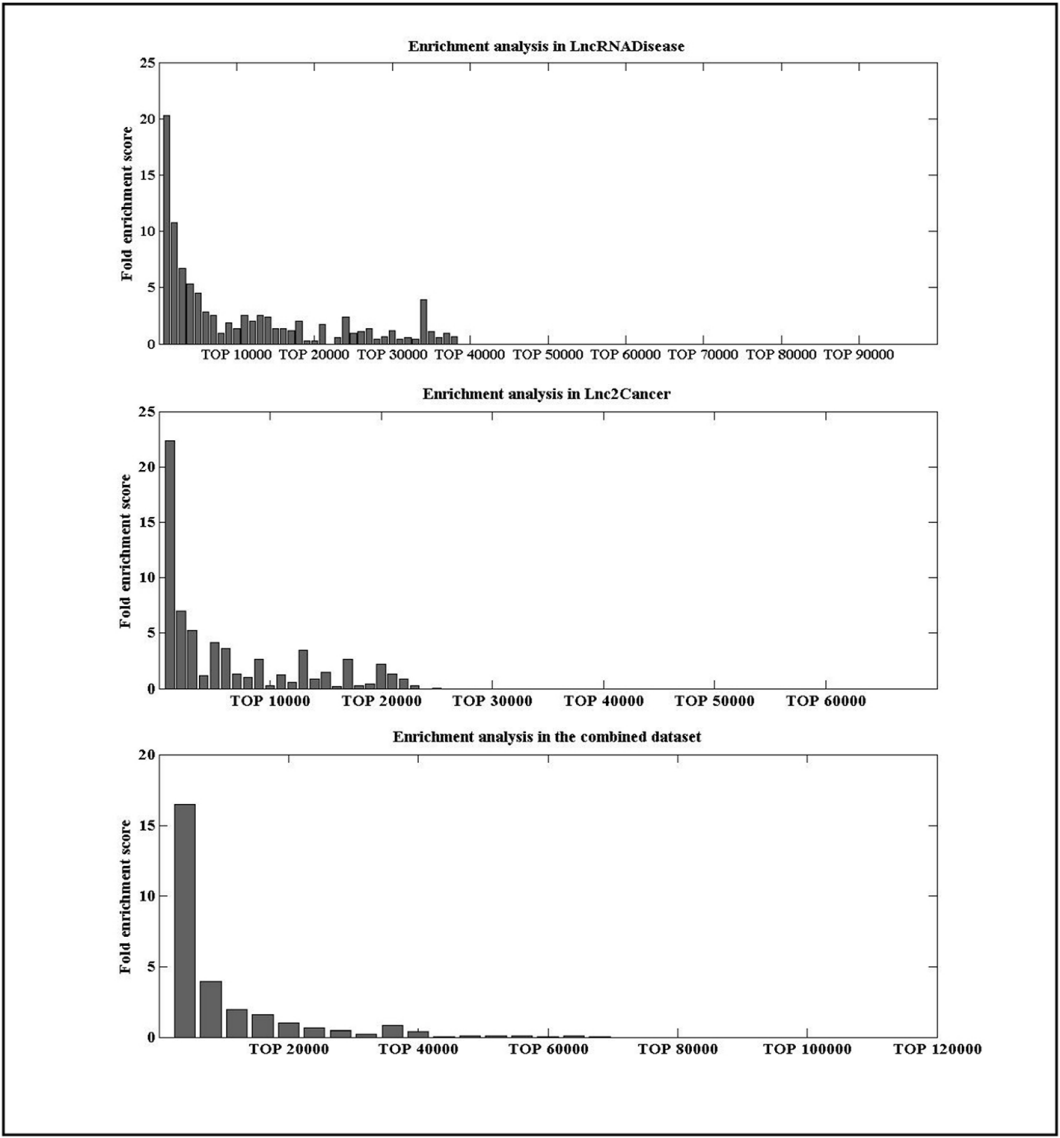
Enrichment analysis in three datasets.

To check whether the lncRNAs with high IDLDA-score were more likely to be disease-related, all candidate lncRNA–disease pairs in two databases were ranked by IDLDA and binned into groups of *x*. Here, we took *x* as 1000 for the data in the LncRNADisease and Lnc2Cancer, and as 10000 for the data in the combined dataset. A fold enrichment score was defined as (mx)/(MN) (Huang et al., 2013), where *m* was the number of distinct experimentally verified associations within one certain bin of *x*, *M* was the number of all distinct experimentally verified lncRNA–disease associations, and *N* was the number of all possible lncRNA–disease associations. For an lncRNA–disease pair, if its fold enrichment score was high for certain bin, it represented this pair was more likely to be related. As shown in **Figure 4**, lncRNAs with high IDLDA-score were more likely to be disease-related in three datasets.

### Case Studies

Case studies were implemented to examine the capability of IDLDA in discovering potential lncRNA–disease associations. For some special diseases, we ranked those candidate lncRNAs based on their corresponding IDLDA-scores. Case studies included three common human diseases (colon cancer, gastric cancer, and breast cancer). Prediction results were verified based on not only the recent updates in the Lnc2Cancer and LncRNADisease but recently published experimental literature. Then we observed the number of the verified lncRNAs in the top 10 and 50 predictions in both databases, all the ranking results have been listed in **Tables 2**–**4**.

**Table 2:** Case study of colon cancer.

**lncRNA**	**Evidence (PMID)**	**Rank (Lnc2Cancer)**	**Rank (LncRNADisease)**
HOTAIR	24667321	1	4
MALAT1	22996375	2	3
MEG3	14602737	3	5
H19	21874233	4	1
ANRIL	23416462	5	14
GAS5	28722800	6	7
UCA1	26885155	7	10
PVT1	29552759	8	6
NEAT1	26552600	11	33
SPRY4-IT1	27621655	16	36
XIST	29679755	23	8
PTENP1	Unconfirmed	36	11

**Table 3:** Case study of breast cancer.

**lncRNA**	**Evidence (PMID)**	**Rank (Lnc2Cancer)**	**Rank (LncRNADisease)**
HOTAIR	24721780	1	4
MALAT1	22492512	2	3
H19	16707459	3	1
MEG3	14602737	4	6
ANRIL	17440112	5	13
UCA1	26439035	6	10
GAS5	29655698	7	7
TUG1	28053623	8	49
PVT1	17908964	9	5
NEAT1	2541770	10	18
XIST	24141629	15	9
HIF1A-AS1	Unconfirmed	16	43
LincRNA-p21	26656491	18	42
SPRY4-IT1	25742952	20	46
LSINCT5	21532345	26	50
PANDAR	26927017	27	20
KCNQ1OT1	26323944	37	38
PCAT1	28989584	39	17
DLEU2	Unconfirmed	45	39
PTENP1	29085464	50	12

**Table 4:** Case study of gastric cancer.

**lncRNA**	**Evidence (PMID)**	**Rank (Lnc2Cancer)**	**Rank (LncRNADisease)**
HOTAIR	29683069	1	4
MALAT1	29162158	2	3
H19	29687854	3	1
MEG3	28975980	4	5
ANRIL	24810364	5	13
UCA1	29723509	6	11
GAS5	27827524	7	7
PVT1	26925791	8	6
NEAT1	27095450	9	33
XIST	29053187	14	9
LincRNA-p21	28969031	20	40
LSINCT5	25694351	21	41
PANDAR	29719612	24	17
KCNQ1OT1	Unconfirmed	26	36
SRA1	Unconfirmed	49	30

Colon cancer is one of the most common malignant tumors in the world (Xue et al., 2015), killing almost seven hundred thousand people every year (Gu et al., 2017), even the disease-specific mortality rate is close to 33% in the developed countries (Han et al., 2015). In 2018, there are 97220 estimated new cases and 50,630 estimated deaths from Colon Neoplasms in U.S. (Siegel et al., 2018). Some associations between colon cancer and lncRNAs have been discovered by biological experiments (Chen et al., 2015), IDLDA can also predict more colon cancer-related lncRNAs. Consequently, all potentially related lncRNAs which ranked top 10 in both databases had been validated by databases and recent experimental literature. Meanwhile, only PTENP1 which ranked top 50 in both databases was not verified. Some research showed that PTENP1 pseudogene may act as “decoy” by protecting PTEN mRNA from binding to common miRNA and allowing expression of the tumor suppressor protein (Li G, et al., 2014). This indicated that PTENP1 was associated with cancer.

Breast cancer is the second leading cause of cancer deaths in women, accounting for 22% of all cancer deaths in women (Donahue and Genetos, 2013; Karagoz et al., 2015). Some researchers announced that a number of lncRNAs are associated with the formation of breast cancer (Meng et al., 2014; Xu et al., 2015). In this paper, we used IDLDA to discover the potential breast cancer-related lncRNAs. From **Table 3**, we could know that all the potential related lncRNAs which ranked top 40 in both databases had been validated. For example, HOTAIR was ranked first in Lnc2Cancer, recent research had confirmed that HOTAIR was strongly expressed in numerous cancers like breast cancer, colorectal cancer, and lung cancer (Gupta et al., 2010; Li G, et al., 2014; Hrdlickova et al., 2014). Only HIF1A-AS1 and DLEU2 in both databases had not been validated by the same resources.

Gastric cancer is the second major reason for cancer-related death in the world (Guo et al., 2014). A myriad of studies has proved that lncRNAs have played crucial roles in the development of gastric cancer (Zhao et al., 2015). It is clear that the associations between breast cancer and HOTAIR, MALAT1, H19, MEG3, ANRIL, UCA1, GAS5, PVT1, NEAT1, XIST, LincRNA-p21, LSINCT5, PANDAR were validated by databases and related literature from **Table 4**. Only KCNQ1OT1 and SRA1 were not confirmed. But there is a potential relationship between SRA1 and breast cancer (Yan et al., 2011), SRA RNA expression is altered during breast tumorigenesis. The semantic similarity between gastric cancer and breast cancer is very large, perhaps future research could explain the relationship between SRA1 and gastric cancer.

## Discussion

According to previous literature, lncRNAs are associated with a mass of diseases. With the emergence of many biological data about lncRNA, it is urgent to design a powerful and effective computing method to predict the underlying disease-related lncRNAs. In this paper, disease semantic similarity, lncRNA functional similarity, disease/lncRNA Gaussian kernel similarity, and lncRNA–disease associations were integrated on a large scale. We developed a computational model named IDLDA, which based on the diffusion model to predict potential lncRNA–disease associations. IDLDA achieved higher AUROC than other methods in the combined dataset. Meanwhile, local cross-validation, enrichment analysis could also show the reliability of the model. Moreover, case studies of colon cancer, breast cancer, and gastric cancer were also implemented, all lncRNAs which ranked top 10 in both databases were verified, only 2, 2, and 1 lncRNAs which ranked top 50 in both databases were not confirmed by databases and related literature. What is more, the results of local cross-validation showed IDLDA can predict not only new disease-related lncRNAs but new lncRNA-related diseases.

Here are the reasons why IDLDA performs better than some aforementioned methods. Firstly, the lncRNA ensemble similarity and disease ensemble similarity can make full use of the information about known lncRNA–disease associations by integrating lncRNA functional similarity, disease semantic similarity, and the Gaussian kernel similarity. Secondly, both disease ensemble similarity and lncRNA ensemble similarity are used in the diffusion process, IDLDA could predict not only new lncRNAs but also new diseases, overcoming some limitations of previous methods. Thirdly, IDLDA as a semi-supervised method is superior to the supervised methods when the data is incomplete. In particular, semi-supervised method could be implemented without any negative lncRNA–disease associations, which are closer to reality. In short, IDLDA will be an important and powerful bioinformatics tool in biomedical research of the lncRNA–disease association prediction, and even disease treatment.

Although IDLDA is effective, this work has several limitations. Firstly, IDLDA contains two parameters, and finding suitable parameters for different datasets is a challenging task. Additionally, some specific lncRNAs are not associated with certain diseases. If this kind of data can be added to the model in the future, it will certainly be helpful to improve the predictive ability. Successfully established models in the other computational fields would inspire the development of lncRNA–disease association prediction. Perhaps we can improve the predictive performance of IDLDA by integrating more information, such as lncRNA–miRNA information (Chen, 2015b) and disease–drug information (Chen et al., 2016).

## Data Availability Statement

Publicly available datasets were analyzed in this study. This data can be found here: http://www.cuilab.cn/lncrnadisease , http://www.bio-bigdata.net/lnc2cancer. 

## Author Contributions

QW conceived the project, developed the prediction method, designed the experiments, implemented the experiments, analyzed the result, and wrote the paper. GY analyzed the result and revised the paper.

## Funding

GY was supported by the National Natural Science Foundation of China under Grant No. 11631014.

## Conflict of Interest

The authors declare that the research was conducted in the absence of any commercial or financial relationships that could be construed as a potential conflict of interest.

## Supplementary Material

Supplementary Table 1 List_of_lncRNA_disease_associations_of_lncRNAdisease.

Supplementary Table 2 Adjacency_matrix_of_Lnc2Cancer.

Supplementary Table 3 Adjacency_matrix_of_LncRNADisease.

Supplementary Table 4 List_of_lncRNA_disease_associations_of_Lnc2Cancer.

Supplementary Table 5 Performance_comparison_of_different_methods.

Supplementary Data Sheet 1 List_of_lncRNA_disease_associations_of_Combined_dataset.
