# Corrigendum: IDLDA: An Improved Diffusion Model for Predicting LncRNA–Disease Associations

**DOI:** 10.3389/fgene.2020.00137

**Published:** 2020-02-26

**Authors:** Qi Wang, Guiying Yan

**Affiliations:** ^1^ Academy of Mathematics and Systems Science, Chinese Academy of Sciences, Beijing, China; ^2^ School of Mathematical Sciences, University of Chinese Academy of Sciences, Beijing, China

**Keywords:** long non-coding RNA, disease, association prediction, computational prediction model, diffusion model

In the original article, there was a mistake in the legend for **Figure 2** published as an explanation of what *N_d_* and *N_l_* represent was missing. The correct legend appears below.

Also in **Figure 2** the diagrams shown in Step 2 are incorrect as the formulas were omitted. The corrected **Figure 2** and its legend appear below. The authors apologize for this error and state that this does not change the scientific conclusions of the article in any way. The original article has been updated.

**Figure 2:**
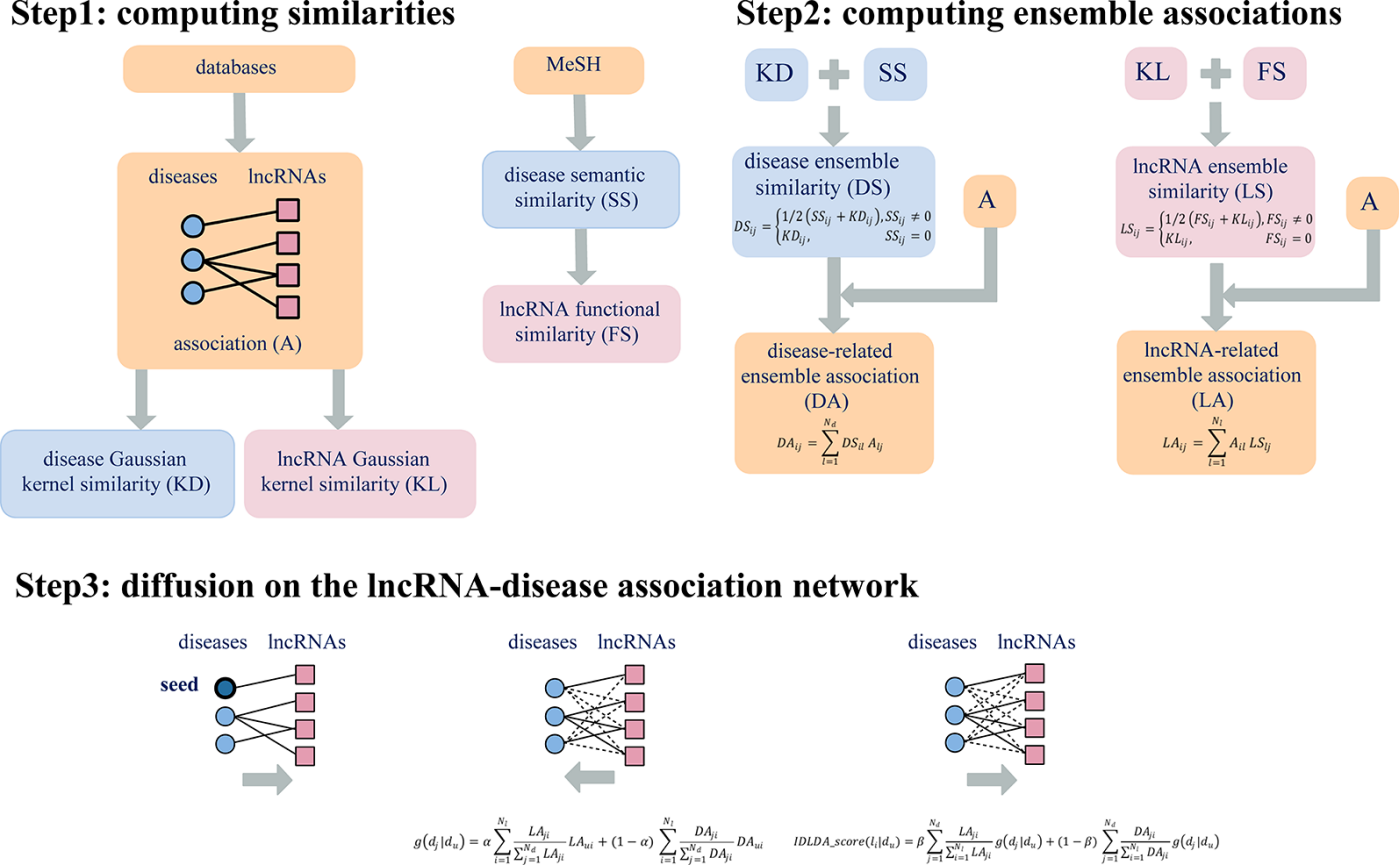
Flowchart of IDLDA. N_d_ and N_l_ represent the number of diseases and the number of lncRNAs, respectively.

